# Chemotherapy Induced Cardiomyopathy: Pathogenesis, Monitoring and Management

**DOI:** 10.4021/jocmr2009.02.1225

**Published:** 2009-03-24

**Authors:** Douraid K. Shakir, Kakil I Rasul

**Affiliations:** a Hamad Medical Corporation, Doha, Qatar. Cardiology and Cardiovascular Dept, Hamad General Hospital; b Haematology/Oncology Dept, Al-Amal Hospital

## Abstract

The survival rate of cancer patients has greatly increased over the last 20 years. However, to achieve this result, a considerable price has been paid in terms of the side effects associated with the intensive anticancer treatment. The most common adverse effect is cardiotoxicity which may compromise the clinical effectiveness of chemotherapy, affecting the patient's survival and quality of life independently of the oncological prognosis. There are 2 types of cardiac toxicities, type I which is more serious and result in permanent damage to the myocardium and type II which is usually reversible. Chemotherapies varies in their incidence of inducing cardiomyopathy, and the onset which may occur acutely (during or shortly after treatment), sub-acutely (within days or weeks after completion of chemotherapy) or chronically (weeks to months after drug administration). Cardiac events associated with chemotherapy may consist of mild blood pressure changes, thrombosis, Electrocardiographic (ECG) changes, arrhythmias, myocarditis, pericarditis, myocardial infarction, cardiomyopathy, cardiac failure (left ventricular failure), and congestive heart failure (CHF). The risk for such effects depends upon: cumulative dose, rate of drug administration, mediastinal radiation, advanced age, younger age, female gender, pre-existing heart disease and hypertension. Serial measurements of LVEF and fractional shortening are the most common indices monitored to assess left ventricular systolic function and cardiotoxicity. This can be achieved by 2-dimensional, M-mode and color Doppler echocardiographic examination; also Cardiac troponins as a biological marker for myocardial damage can be used for monitoring in patients received anthracyclines. Angiotensin-converting enzyme (ACE) inhibitors (ACEIs) have been shown to slow the progression of left ventricular dysfunction in several different clinical settings, including anthracycline-induced cardiomyopathy. Carvedilol and probably with anti-oxidants like Probucol and vitamin E benefits also.

**Keywords:**

Anthracyclines; Cardiomyopathy; Chemotherapy

## Introduction

Chemothearpies are highly effective in treating most cancers, their use is limited by the potential for cardiotoxicity. All these drugs have a wide range of adverse effects the most serious one is cardiotoxicity, these severity of these effects are related to the chemotherapy regimens, patient populations and duration. The occurrence of clinical heart failure seems to be in the range of 1% to 5%, and asymptomatic decrease in left ventricular function is in the range of 5% to 20%. Toxicity can occur early (within 1 year) or late (particularly among children, where late cardiac abnormalities are detectable in two thirds of surviving patients).

Many trials address the role of ACE inhibitors and beta-blockers, effective therapies for established LVSD, in preventing chemotherapy-induced cardiotoxicity. In this article we discuss the types of the cardiomyopathy, diagnosis, prognosis, prevention and managements.

## Chemotherapy induced cardiomyopathy

The survival rate of cancer patients has greatly increased over the last 20 years. However, to achieve this result, a considerable price has been paid in terms of the side effects associated with the intensive anticancer treatment. Cardiotoxicity may compromise the clinical effectiveness of chemotherapy, affecting the patient's survival and quality of life independently of the oncological prognosis. As a result of the increasing number of long-term cancer survivors and of the tendency to use higher doses of cytotoxics and combined treatments with synergistic cardiotoxic effects, the magnitude of this problem is growing. Accordingly the onset of cardiac dysfunction, even if it is asymptomatic, not only negatively affects cancer patients' cardiac outcomes, but also seriously limits their therapeutic opportunities. There are 2 types of cardiac toxicities, type I which is more serious and result in permanent damage to the myocardium and type II which is usually reversible. Features and risk factors of both types summarised in Table 1. Anthracyclines, even after three decades, continue to play a prominent role in the treatment of a wide variety of both hematologic and solid tumors; it is now well established that anthracycline cardiotoxicity is a cumulative dose-related effect, suggesting that each administration constitutes additive or sequential damage.

**Table 1:** Chemotherapy related cardiac dysfunction

TYPE I e.g. Doxorubicin	TYPE II e.g. Trastuzumab
Cellular death Damage starts with first administration	Cellular dysfunction
Biopsy changes	No typical biopsy changes
Cumulative dose related	Not cumulative dose related
Permanent damage (Myocyte death)	Predominately reversible (Myocyte dysfunction)
Risk factors	Risk factors
Prior/concurrent radiotherapy	Prior/concurrent anthracycline
Combination chemotherapy	Paclitaxel
Age	Age
Previous cardiac disease	Previous cardiac disease
Hypertension	Obesity (BMI > 25)

As early as 1967, there were reports of heart failure in children treated with doxorubicin for leukemia; from that time concerns regarding chemotherapy starts. Aggressive and combination chemotherapy has achieved remission in most types of cancers. However, concerns for, or manifestations of, cardiac adverse events may result in discontinuation of or reluctance to use a particular agent at an effective dose. Cytostatic antibiotics of the anthracycline class have been clearly associated with cardiotoxicity. However, there are a number of other chemotherapy agents that cause cardiotoxicity and yet are not well recognized Cardiac events associated with chemotherapy vary in incidence and may occur acutely (during or shortly after treatment), sub-acutely (within days or weeks after completion of chemotherapy) or chronically (weeks to months after drug administration). They may also occur as late squeal, many years after the end of treatment. Cardiac events associated with chemotherapy may consist of mild blood pressure changes, thrombosis, Electrocardiographic (ECG) changes, arrhythmias, myocarditis, pericarditis, myocardial infarction, cardiomyopathy, cardiac failure (left ventricular failure), and congestive heart failure (CHF). The substantial limitations of using only changes in LVEF are compromised further by our knowledge that approximately half of all heart failure occurs in patients who maintain a normal LVEF; their overall cardiac outcomes are similar to those who exhibit a low LVEF [1].

Cardiotoxicity may depend on the dose administered during each course or on the total cumulative dose, or may be completely independent of the dose like Anthracycline-induced cardiotoxicity which has been recognized for more than 20 years. It has been described as 3 distinct types of cardiotoxicity. Acute or sub-acute injury is a rare form of cardiotoxicity that may occur immediately after a single dose or a course of anthracycline therapy, with clinical manifestations occurring within a week of treatment. These may be in the form of transient electrophysiological abnormalities, a pericarditis, myocarditis syndrome or acute left ventricular failure. The electrophysiological abnormalities may present as nonspecific ST and T wave changes, T wave flattening, decreased QRS voltage and prolongation of QT interval. Sinus tachycardia is the most common rhythm disturbance. ECG changes may be seen in 20 to 30% of the patients [2]. Arrhythmias, including ventricular, supraventricular and junctional tachycardia, are seen in 0.5 to 3% of patients with an overall incidence of 0.7% [2]. More serious arrhythmias, such as atrial flutter or atrial fibrillation, are rare. Sub-acute cardiotoxicity has resulted in acute failure of the left ventricle, pericarditis or a fatal pericarditis-myocarditis syndrome in some rare cases. The ECG changes or arrhythmias do not seem related to chronic cardiomyopathy.

Early onset chronic progressive cardiotoxicity: anthracyclines can also induce early onset progressive chronic cardiotoxicity resulting in cardiomyopathy. This is a more common and clinically important type of cardiotoxicity [3].Chronic anthracycline-induced cardiomyopathy usually presents within a year of treatment. It may persist or progress even after discontinuation of anthracyclines therapy, and may evolve into a chronic dilated cardiomyopathy in adult patients and restrictive cardiomyopathy in pediatric patients [4]. Late onset chronic progressive anthracycline cardiotoxicity causes ventricular dysfunction [5], heart failure and arrhythmias [6] years to decades after chemotherapy has been completed. This suggests that patients who have received anthracyclines chemotherapy and survived their cancer may have undetected increases in morbidity and mortality due to cardiotoxicity. There may be a period of time, after completion of treatment, during which patients may experience no symptoms of left ventricular dysfunction or arrhythmia and cardiac function may appear normal. After the initial acute myocardial insult, there is a progressive decrease in ventricular function leading to late onset decompensation. An increased incidence of severe echocardiographic abnormalities has been seen with increased duration of follow-up. An 18% incidence of reduction in fractional shortening on resting echocardiogram was observed 4 to 10 years after completion of anthracycline therapy [6]. Cumulative doses of doxorubicin as low as 228 mg/m^2^ have shown to increase after-load or decrease contractility, or both, in 65% of patients with leukaemia up to 15 years after treatment with anthracyclines [7]. Late onset arrhythmia and sudden death has occurred more than 15 years after anthracycline treatment [8]. This could mean that more anthracyclines induced cardiotoxicity may appear in the future in patients who are presently asymptomatic. Patients may remain in a compensated state for many years until stressors such as acute viral infection [9] or cardiovascular stressors such as weight lifting, pregnancy and surgery [6] could possibly trigger a cardiac event.

## Pathogenesis

The cause of anthracycline-induced cardiotoxicity is probably multi-factorial. Free radicalmediated myocyte damage is one of the most thoroughly studied mechanisms by which anthracyclines have been proposed to cause cardiotoxicity [10]. The myocardium is more susceptible to free radical damage than other tissues because it has comparatively less superoxide dismutase and catalase activity, and its major defense against free radical damage, glutathione peroxidase, is suppressed by doxorubicin. The superhydroxide free radicals accumulate and cause severe lipid peroxidation, leading to extensive destruction of the mitochondrial membranes, endoplasmic reticulum and nucleic acid. Circulating pro-inflammatory cytokines have also been implicated in anthracycline cardiotoxicity. Doxorubicin induces the release of histamine and tumour necrosis factor-α from macrophages and interleukin-2 from monocytes [11]. These cytokines have functional receptors on the myocardium and their release may result in dilated cardiomyopathy. Adrenergic dysfunction and down regulation of myocardial histamine and β-adrenergic receptors has also been proposed as a cause for an evolving and established anthracyclineinduced ventricular dysfunction.

## Risk factors for cardiotoxicity

Some of the risk factors relating to early and late (but not acute) cardiotoxicity have been reported. These include cumulative dose, rate of drug administration, mediastinal radiation, advanced age, younger age, female gender, pre-existing heart disease and hypertension. A multivariate analysis of these factors based on histological evidence of anthracycline-induced cardiac damage concluded that higher rates of administration and previous cardiac irradiation were independent risk factors. At a cumulative total dose of < 400 mg/m^2^ body surface area, the incidence of CHF was found to be 0.14%. This increased to 7% at a dose of 550 mg/m^2^ and to 18% at a dose of 700 mg/m^2^ [4]. There is a formula to calculate the cardiac toxicity as follow Y= (X)^2^/a, Y is the likelihood of developing congestive heart failure, X equals to number of cycles of anthracycline-containg regimen administered, a equals to correction constant determined by cycle dose and the duration between the cycles, so if a patient receives 9 cycle of anthracycline in a dose of 50 mg/m^2^ every 21 days, so it will be Y= 81/16 X100, the Y will be equal to 5%, this is the risk of developing congestive heart failure [4].

Serial and post-therapy cardiac monitoring is necessary to reduce morbidity due to anthracycline-induced cardiotoxicity. Patients should be monitored for clinical signs of cardiomyopathy by physical examination, chest x-rays, ECG, echocardiogram, endomyocardial biopsy if feasible and radionuclide angiography before initiation of treatment and at periodic intervals during therapy. Physical examination alone may miss over 50% of cases of early and reversible chemotherapy-induced CHF [12]. Acute ECG changes and arrhythmias following doxorubicin therapy occur in 0 to 14% of patients. Serial measurements of LVEF and fractional shortening are the most common indices monitored to assess left ventricular systolic function and cardiotoxicity. This can be achieved by 2-dimensional, M-mode and color Doppler echocardiographic examination. Baseline LVEF estimation is recommended before the start of doxorubicin therapy. If LVEF is ≤ 30%, starting chemotherapy is not recommended. Patients with LVEF ≥ 30% but <50% can receive doxorubicin, but measurements should be repeated before each dose. For patients with baseline LVEF ≥ 50%, evaluations should be repeated after a cumulative dose of 250 to 300 mg/m^2^ and thereafter at 450 mg/m^2^ if they have no risk factors. If patients have known cardiovascular disease, prior radiation treatment to the chest, abnormal ECG changes or concomitant cardiotoxic chemotherapy, LVEF measurement should be repeated at 400 mg/m^2^ instead of 450 mg/m^2^. It should be monitored with each dose thereafter. Doxorubicin therapy should be stopped if there is a ≥ 10% absolute drop in the ejection fraction associated with a decrease in LVEF to ≤ 50% in patients with baseline LVEF ≥ 50%, and to ≤ 30% in patients with baseline LVEF < 50% but > 30% [13].

Biomarkers such as B-type natriuretic peptide and troponins (I and T) are increasingly being used to stratify patients into higher and lower risk categories. This process is well established in the cardiology literature and recently has been reported in oncology patients. In fact, an elevated troponin during chemotherapy seems to correlate with increased risk for the development of cardiac toxicity [14]. Cardiac troponins as a biological marker for myocardial damage can be used for monitoring in patients received anthracyclines. troponin I (TnI) soon after high-dose chemotherapy (HDC) is a strong predictor of left ventricular dysfunction and poor cardiac outcome, particularly in patients showing a persistent TnI increase [15].

Some cardio-protective agents is used which associated with a decrease in cardiotoxicity and facilitates the use of higher cumulative doses of anthracyclines but it is beyond our article. Also Cardiotoxicity of anthracyclines can be minimized by using analogues that may be less cardiotoxic. Compounds such as epirubicin and idarubicin exhibited decreased cardiotoxicity in preclinical trials.

The use of anti-oxidant agents or iron chelators finds their way in the prevention for cardiac toxicities. Probucol, vitamin E (as anti-oxidants) and carvedilol have shown promise in animal studies.

Angiotensin-converting enzyme (ACE) inhibitors (ACEIs) have been shown to slow the progression of left ventricular dysfunction in several different clinical settings, including anthracycline-induced cardiomyopathy [16]. Furthermore, data referring to experimental models suggest that the cardiac renin-angiotensin system (RAS) plays an important role in the development of anthracycline-induced cardiomyopathy and that treatment with ACEIs protects against chemotherapy-induced cardiotoxicity. In a recent study showed that [17] early treatment with enalapril in patients with evidence of myocardial cell injury (TnI increase) after HDC seems to prevent the development of cardiotoxicity and the occurrence of associated adverse clinical events. The benefits of and clinical indications for ACEIs have been clearly defined in many cardiovascular conditions such as chronic heart failure, asymptomatic left ventricular dysfunction, acute myocardial infarction, and hypertension and in patients at increased risk of cardiovascular events. In cardiomyopathy, because of anthracycline induced cardiotoxicity, the use of enalapril has been proved to be beneficial in prolonging survival and in preventing further deterioration of cardiac function.

At the end, frequent monitoring and follow up for patients took anthracyclines with troponins and echocardiograms with early administration of carvedilol, enalapril and probably with anti-oxidants like Probucol and vitamin E will benefit.
